# Supplementary material for: Virus Etiology, Diversity and Clinical Characteristics in South African Children Hospitalised with Gastroenteritis
Source: Viruses. 2021 Jan 30;13(2):215. doi: 10.3390/v13020215 (PMC7911269; doi:10.3390/v13020215)
Supplement: Supplementary file 1 [file viruses-13-00215-s001.pdf]

Supplementary Table S1: Primers used for singleplex detection of norovirus and genotyping of norovirus, sapovirus, rotavirus and *FUT2*

**Virus detection**	**Primer/Probe**	**Sequence (5′ - 3′)#**	**Nucleotide Position**	**Product Size**
Norovirus GI†	QNIF4	CGCTGGATGCGNTTCCAT	5291–5308	85 bp
	NV1LCR	CCTTAGACGCCATCATTTAC	5354–5376	
	Probe: Norovirus GI	FAM-TGGACAGGAGAYCGCRATG - TAMRA	5321–5340	
Norovirus GII‡	QNIF2	ATGTTCAGRTGGATGAGRTTCTCWGA	5012–5037	88 bp
	COG2R	TCGACGCCATCTTCATTCACA	5080–5100	
	Probe: QN1FS	FAM -AGCACGTGGGAGGGCGATCG - TAMRA	5042–5061	
**Norovirus amplification**	**Primer Name**	**Sequence (5′ – 3′)**		**Product Size**
	**(polarity)**			**(bp)**
**GI†**				
Region AC	JV12Y	ATACCACTATGATGCAGAYTA	4279–4299	~1.39 kb
	G1SKR (-)	CCAACCCARCCATTRTACA	5653-5671	
Region BC	MON432	TGGACICGYGGICCYAAYCA	5093-5112	578 bp
	G1SKR (-)	CCAACCCARCCATTRTACA	5653-5671	
Region C	G1SKF	CTGCCCGAATTYGTAAATGA	5342-5361	329 bp
	G1SKR (-)	CCAACCCARCCATTRTACA	5653-5671	
**GII‡**				
Region AC	JV12Y	ATACCACTATGATGCAGAYTA	4279–4299	~1.1 kb
	G2SKR (-)	CCRCCNGCATRHCCRTTRTACAT	5367–5389	
Region BC	MON431	TGGACIAGRGGICCYAAYCA	4820-4839	569 bp
	G2SKR (-)	CCRCCNGCATRHCCRTTRTACAT	5367–5389	
Region C	G2SKF	CNTGGGAGGGCGATCGCAA	5046–5064	343 bp
	G2SKR (-)	CCRCCNGCATRHCCRTTRTACAT	5367–5389	
**Sapovirus* amplification**	**Primer/probe**	**Sequence (5'-3')**		
First round	SV-F13	GAYYWGGCYCTCGCYACCTAC	5074–5094	802 bp
	SV-F14	GAACAAGCTGTGGCATGCTAC	5074–5094	
	SVR-DS3 (-)	GGTGAVAVMCCATTYTCCAT	5857–5876	
	SVR-DS4 (-)	GGHGAHATNCCRTTBYSCAT	5857–5876	
Second round	SaV1245Rfwd	TAGTGTTTGARATGGAGGG	5159–5177	339 bp
	SVR-DS5 (-)	CCCCACCCKGCCCACAT	5482–5498	
	SVR-DS6 (-)	CCCCAMCCMGCMMACAT	5482–5498	
**VP type**	**Primer**	**Sequence 5'-3'**		
VP7	sBeg9	GGCTTTAAAAGAGAGAATTTC	1-21	1062 bp
	End 9 (-)	GGTCACATCATACAATTCTAATCTAAG	1036-1062	
	9con1	TAGCTCCTTTTAATGTATGG	37-56	902 bp
	EndA (-)	ATAGTATAAAATACTTGCCACCA	922-944	
VP4	Con3	TGGCTTCGCTCATTTATAGACA	11-32	876 bp
	Con2 (-)	ATTTCGGACCATTTATAACC	868-887	
	VP4F	TATGCTCCAGTNAATTGG	132-149	663 bp
	VP4R (-)	ATTGCATTTCTTTCCATAATG	775-795	

FUT2 Primers	Primers	Sequence 5'-3'		Product Size
	FUT2Ex2F	ACACACCCACACTATGCCTGCAC		1 263 bp
	FUT2Ex2R (-)	ACTTGCAGCCCAACGCATCTT		

#IUPAC codes used to indicate degenerate primers. Primer positions based on GenBank accession numbers †M87991, ‡X86557, *AY237422.

Supplementary Table S2: Cycling parameters for the amplification of the genotyping regions of norovirus, rotavirus, sapovirus and the *FUT2 gene*.

**Virus amplification**	**Norovirus Round 1**	**Norovirus Round 2**	**Rotavirus (Round 1-2)**	**Sapovirus (Round 1)**	**Sapovirus (Round 2)**	***FUT2***
Kit used	EmeraldAmp MAX HS PCR (Thermo Scientific, Waltham, MA)		GoTaq Hotstart Polymerase (Promega)	EmeraldAmp MAX HS PCR		Q5® Hot Start High-Fidelity DNA Polymerase (NEB)
Initial denaturation	95°C, 10 min	95°C, 10 min	95°C, 1 min	95°C, 10 min	95°C, 10 min	98°C, 30 sec
Denaturation	94°C, 30 sec	94°C, 30 sec	95°C, 1 min	94°C, 30 sec	94°C, 30 sec	98°C, 30 sec
Annealing	50°C, 30 sec	55°C, 30 sec	42°C, 1 min	50°C, 30 sec	50°C, 30 sec	66°C, 30 sec
Extension	72°C, 1 min/kb	72°C, 1 min/kb	72°C, 1 min	72°C, 1 min/kb	72°C, 1 min/kb	72°C, 90 sec
Cycles	40	40	35	40	45	35
Final extension	72°C, 10 min	72°C, 10 min	72°C, 7 min	72°C, 10 min	72°C, 10 min	72°C, 2 min

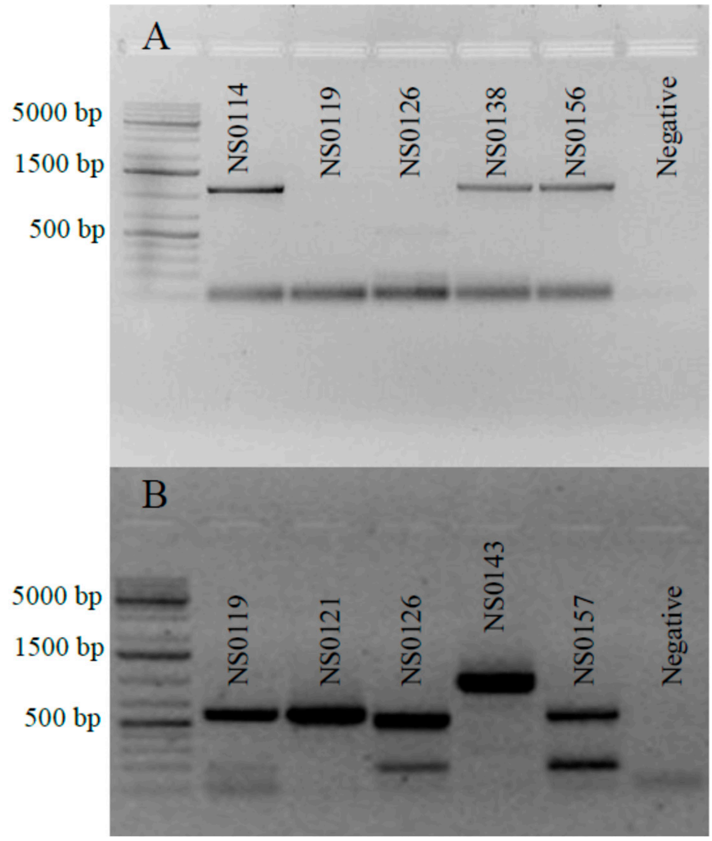

Supplementary Figure S1: Amplification of A) Region AC (~1100 bp) and B) Region BC (~560 bp) of norovirus from various strains detected in hospitalised children. The gel shows representative amplification products. In some cases, only non-specific products amplified (NS0143) or a combination of specific and non-specific amplified (NS0126, NS0157). Amplicons were purified and sequenced.

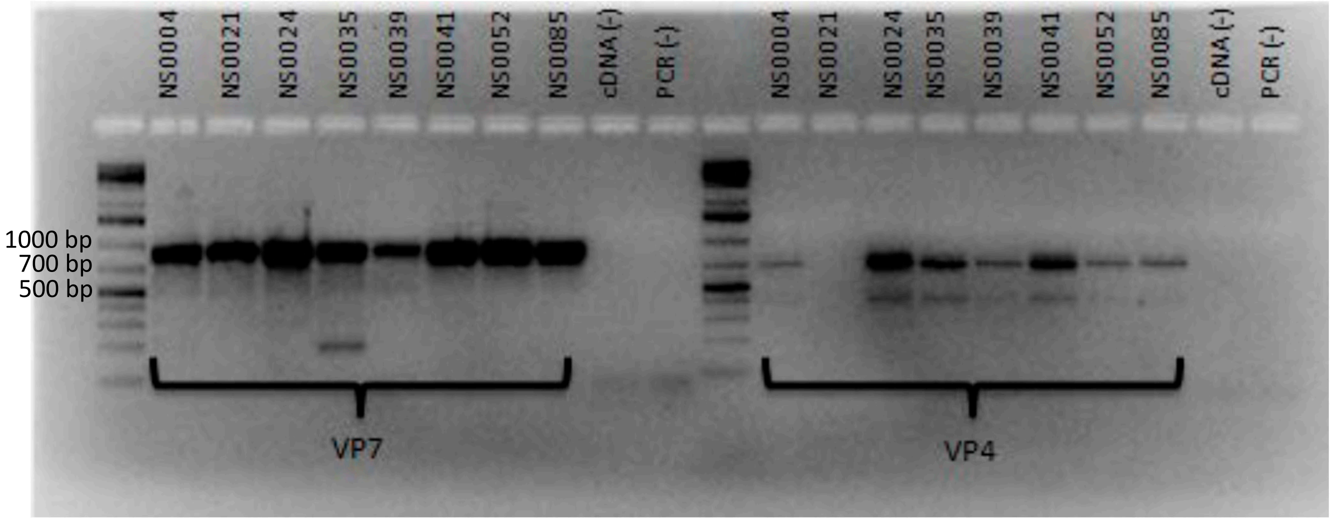

Supplementary Figure S2: Amplification of VP7 (902 bp) and VP4 (663 bp) of rotavirus from various strains detected in hospitalised children. The gel shows representative amplification products. Amplicons were purified and sequenced.

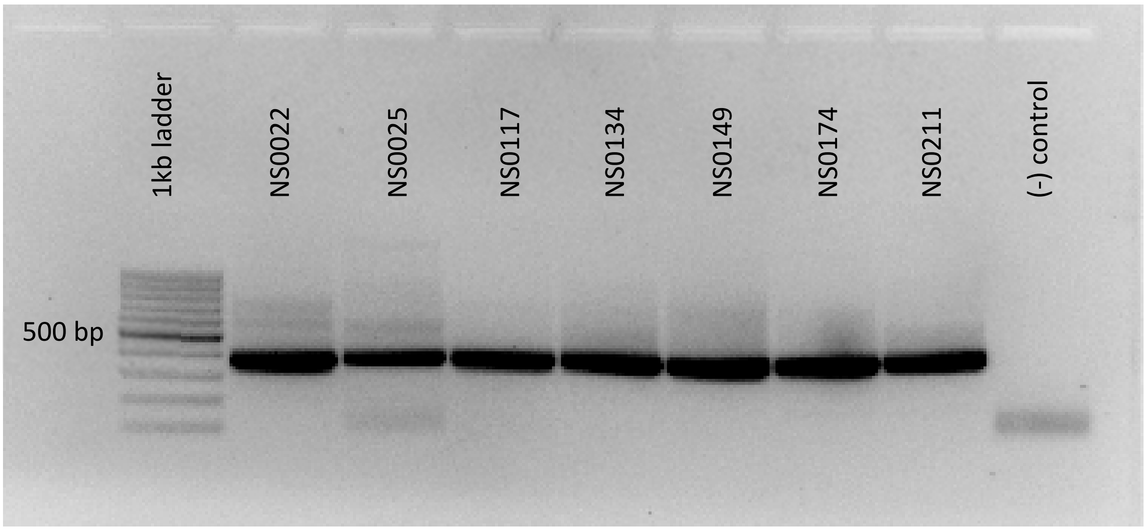

Supplementary Figure S3: Amplification of the partial sapovirus capsid (~339 bp) from various strains detected in hospitalised children. The gel shows representative amplification products. Amplicons were purified and sequenced.
